# Structure–Activity Relationship of Oxyphosphonate Inhibitors: Role of Heteroatoms in Controlling Pitting Corrosion of Ferritic–Martensitic Steel EP-450

**DOI:** 10.3390/molecules31142504

**Published:** 2026-07-17

**Authors:** Tolganay Y. Zharkynbek, Dana Askar, Raushan B. Koizhaiganova, Kira V. Tsay, Khaidar S. Tassibekov, Tulegen M. Seilkhanov, Ilya G. Shenderovich, Valentina K. Yu

**Affiliations:** 1 Laboratory of Synthetic and Natural Medicinal Compounds Chemistry, A.B. Bekturov Institute of Chemical Sciences, 106 Sh. Ualikhanov St., Almaty 050010, Kazakhstan; danaaskarova002@gmail.com (D.A.); k_raushan@yahoo.com (R.B.K.); kh.tassibekov@ihn.kz (K.S.T.); 2 Faculty of Chemistry and Chemical Technology, Al-Farabi Kazakh National University, 71/23 Al-Farabi Ave., Almaty 050040, Kazakhstan; 3 Laboratory of Ion-Plasma Technology, Institute of Nuclear Physics, 1 Ibragimov St., Almaty 050032, Kazakhstan; kira.tsai7@gmail.com; 4 Laboratory of Engineering Profile NMR Spectroscopy, Sh. Ualikhanov Kokshetau University, 76 Abai St., Kokshetau 020000, Kazakhstan; tseilkhanov@mail.ru; 5 Faculty of Chemistry and Pharmacy, University of Regensburg, Universitaetstrasse 31, 93053 Regensburg, Germany

**Keywords:** structure–activity relationship, oxyphosphonate, pitting corrosion, heteroatom substitution, nitrogen vs. sulfur effect, corrosion inhibition efficiency, Fe-ligand coordination

## Abstract

The structure–activity relationship of three oxyphosphonate inhibitors differing in heteroatom type (C, N, S) was examined to clarify their influence on the pitting corrosion resistance of ferritic–martensitic steel EP-450 in chloride media. Gravimetric tests in 10% FeCl_3_, supported by surface microscopy and adsorption analysis, showed that EP-450 is highly susceptible to localized attack, with pits nucleating preferentially at carbide-enriched, chromium-depleted regions. Addition of dimethyl(1-hydroxycyclohexyl)phosphonate reduced the corrosion rate from 49 to 33 mm/year at 2.0 g/L, corresponding to ≈33% protection, while the nitrogen-containing dimethyl[1-(2-ethoxyethyl)-4-hydroxypiperidin-4-yl]phosphonate produced the largest decrease in mass loss, exceeding a 55% reduction under identical conditions. The sulfur-bearing dimethyl(4-hydroxytetrahydro-2H-thiopyran-4-yl)phosphonate afforded an intermediate effect. Adsorption analysis for the cyclohexyl derivative suggested mixed physisorption–chemisorption with limited surface coverage, while heteroatom substitution (N or S) is consistent with a change in adsorption configuration and interfacial packing that can yield a more compact protective layer. The observed inhibition efficiency increased in the sequence C < S < N, which is interpreted empirically in terms of heteroatom-dependent adsorption geometry and film integrity rather than conjugation-driven activation of the P=O group.

## 1. Introduction

Improving the corrosion resistance of structural materials remains a critical challenge during both operation and post-irradiation storage of components in fast nuclear reactors. Ferritic–martensitic steel EP-450 is widely used for reactor assemblies because of its high strength, thermal stability, and radiation tolerance, yet long-term exposure to aqueous environments in spent-fuel pools can induce localized corrosion and microstructural degradation [1]. Such damage is typically associated with pit initiation near carbide-enriched regions and chromium-depleted zones, which act as preferential anodes under oxidizing chloride conditions, as shown in irradiated ferritic–martensitic steels, where microstructural defects and carbide-associated regions critically influence material response [2]. Previous studies have shown that nitrogen alloying can improve passive-film stability and delay pit propagation by promoting Cr–N interactions within the protective oxide [3,4]. Nevertheless, in the case of EP-450, corrosion susceptibility after neutron irradiation or extended aqueous storage remains a major concern, emphasizing the need for advanced molecular inhibitors capable of stabilizing the surface and mitigating localized electrochemical attack [1].

**Figure 1:**
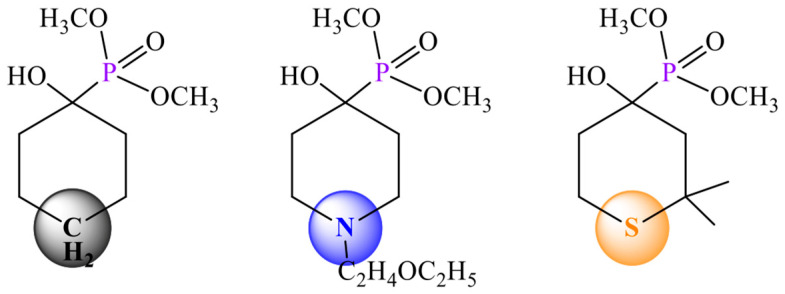
Molecular structures of the three oxyphosphonates studied as corrosion inhibitors.

Traditional inorganic inhibitors based on Zn^2+^, Ni^2+^, or Cu^2+^ ions can reduce corrosion rates but raise environmental and disposal concerns [5,6]. Organophosphonate derivatives have therefore gained considerable attention as environmentally benign alternatives [7,8]. These compounds exhibit high hydrolytic stability, low toxicity, and strong affinity for metallic surfaces through adsorption of P=O and P-OH functional groups, which can promote the formation of interfacial films that hinder chloride penetration and help stabilize the Fe^2+^/Cr^3+^ oxide network [9]. Recent investigations have shown that corrosion inhibition performance depends strongly on the presence and identity of electron-rich heteroatoms within the organic backbone, such as oxygen, nitrogen, and sulfur, which increase absorption properties of the inhibitor on metal surfaces [10,11]. To illustrate the molecular differences among the oxyphosphonates examined in this work, the structural motifs containing O, N, and S heteroatoms are shown in Figure 1, highlighting the donor groups responsible for surface coordination. Variations in donor capability among these heteroatoms provide a plausible basis for comparing their adsorption behavior and corrosion-mitigation performance on ferritic–martensitic steel EP-450.

Although the influence of heteroatom substitution has been explored for mild and low-alloy steels, its mechanistic role in high-chromium ferritic–martensitic alloys remains poorly understood. The microstructural complexity of EP-450, characterized by carbide segregation and Cr-depleted regions, amplifies susceptibility to pit initiation and growth, making it a useful model system for examining structure–activity correlations in heteroatom-functionalized inhibitors.

We hypothesize that heteroatom substitution (N or S) may modify the adsorption configuration and surface packing of oxyphosphonates on EP-450 by introducing an auxiliary donor site in addition to phosphonate oxygen atoms, thereby potentially affecting film compactness and resistance to chloride-induced breakdown. To verify this hypothesis, three oxyphosphonate molecules, dimethyl(1-hydroxycyclohexyl)phosphonate, dimethyl[1-(2-ethoxyethyl)-4-hydroxypiperidin-4-yl]phosphonate, and dimethyl(4-hydroxy-tetrahydro-2H-thiopyran-4-yl)phosphonate, were comparatively evaluated under chloride-induced pitting conditions. Gravimetric corrosion measurements combined with post-exposure surface observations were employed to evaluate the inhibition performance of the investigated oxyphosphonates. Based on this framework, a comparative assessment of oxyphosphonate inhibitors differing by heteroatom nature was undertaken to establish a structure-activity relationship controlling the localized corrosion resistance of ferritic-martensitic steel EP-450. Accordingly, this work is positioned as a comparative structure–activity investigation aimed at identifying trends in inhibition performance under aggressive chloride conditions, with mechanistic implications discussed cautiously.

## 2. Results and Discussion

The present study was designed as an initial comparative structure–activity evaluation of oxyphosphonate inhibitors under accelerated ferric-chloride pitting conditions. It should be emphasized that the present study is based on gravimetric measurements and post-exposure optical observations, which provide a macroscopic assessment of corrosion behavior. These methods do not directly resolve electrochemical kinetics or molecular-level adsorption mechanisms. Therefore, interpretations related to adsorption configuration, heteroatom coordination, and film compactness are presented as plausible hypotheses consistent with observed trends rather than direct experimental confirmation. Future studies incorporating electrochemical (EIS, polarization) and surface-sensitive techniques (SEM, XPS) are required for mechanistic validation.

**Figure 2:**
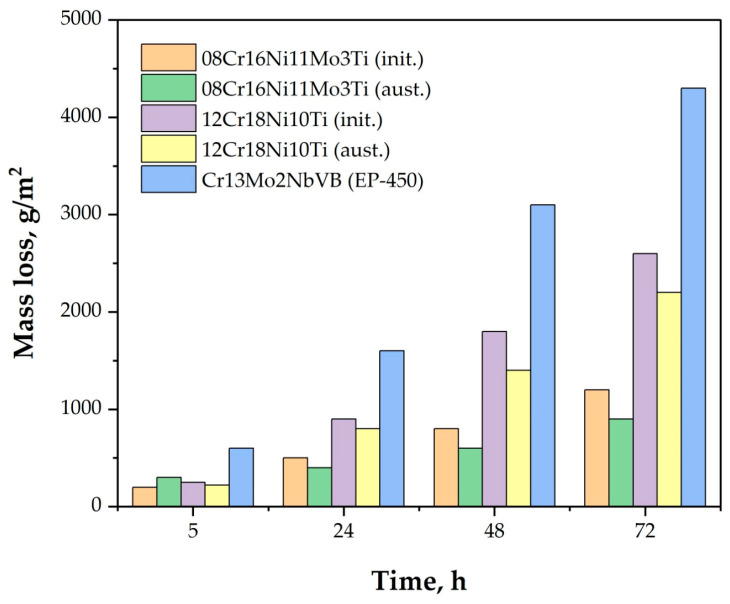
Mass loss of stainless steels in 10% FeCl_3_·6H_2_O at 20–23 °C.

*Pitting characteristics of EP-450 steel.* In the absence of an inhibitor, the corrosion damage exhibits a strongly localized pattern. Pitting sites form mainly along grain boundaries and in regions enriched with carbide particles of the M_23_C_6_ type (Cr, Fe, Ni) and NbC. These zones likely display increased chemical reactivity because of chromium depletion, which may promote dissolution of the metallic matrix. Previous studies report similar behavior for ferritic–martensitic steels exposed to chloride environments [12,13]. The observed pitting morphology in this study is consistent with this mechanism and indicates that the martensitic ferritic structure of EP-450 is highly susceptible to localized attack under chloride exposure, as shown in Figure 2.

### 2.1. Corrosion Behavior of EP-450 Steel in the Absence of Inhibitor

Figure 2 summarizes the comparative mass-loss behavior of several stainless steels exposed to 10% FeCl_3_·6H_2_O. Among all alloys, the ferritic–martensitic steel EP-450 (Cr13Mo2NbVB) demonstrates the highest mass loss throughout the entire immersion period, indicating markedly lower pitting-corrosion resistance compared with austenitic grades. This response is consistent with earlier reports showing that ferritic–martensitic steels are particularly vulnerable to chloride-induced passivity breakdown under oxidizing conditions [14,15,16].

**Figure 3:**
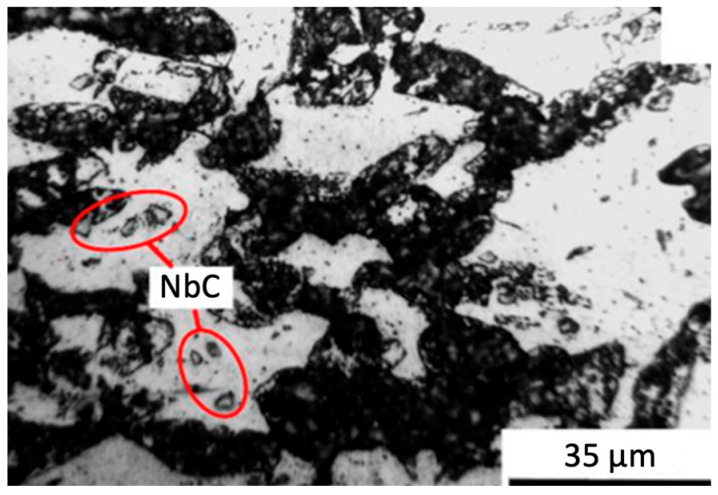
Surface morphology of EP-450 steel after exposure to 10% FeCl_3_ solution (Jeol 2100, optical microscopy, reflected light, ×200 magnification).

To further understand the degradation mechanism, EP-450 was selected as the focus material. After only 5 h of exposure, the steel exhibits numerous pits across the surface (Figure 3), revealing an early breakdown of the passive film. The dark zones correspond to well-developed pitting cavities, which preferentially nucleate within martensitic grains enriched in carbide precipitates such as M_23_C_6_ (Cr-Fe-Ni) and NbC. These zones may display increased chemical reactivity due to possible chromium depletion near carbide precipitates, which promotes dissolution of the metallic matrix. Previous studies report similar behavior for ferritic–martensitic steels exposed to chloride environments [2,17,18]. The observed pitting morphology in this study supports this mechanism and indicates that the martensitic ferritic structure of EP-450 is highly susceptible to localized attack under chloride exposure, as shown in Figure 3. However, the optical microscopy data were used only for qualitative confirmation of localized corrosion. Quantitative pit morphology parameters, including pit depth, pit density, and pit area fraction, were not determined in the present study. Therefore, the gravimetric results should be interpreted as indicators of overall pitting severity rather than as direct quantitative descriptors of pit nucleation and growth.

The preferential attack near carbide-rich regions aligns with established models of micro-galvanic activation, in which chromium-depleted zones adjacent to carbides exhibit lower resistance to chloride-induced depassivation [19,20]. The observed particles are attributed to carbide phases (e.g., M_23_C_6_ and NbC) based on their characteristic morphology and prior literature reports on ferritic–martensitic steels. However, local EDS elemental mapping was not performed in the present study; therefore, chromium depletion near carbide precipitates is discussed as a plausible literature-supported mechanism rather than as directly confirmed experimental evidence. NbC particles may also act as cathodic sites, intensifying local anodic dissolution and facilitating pit initiation [21].

**Figure 4:**
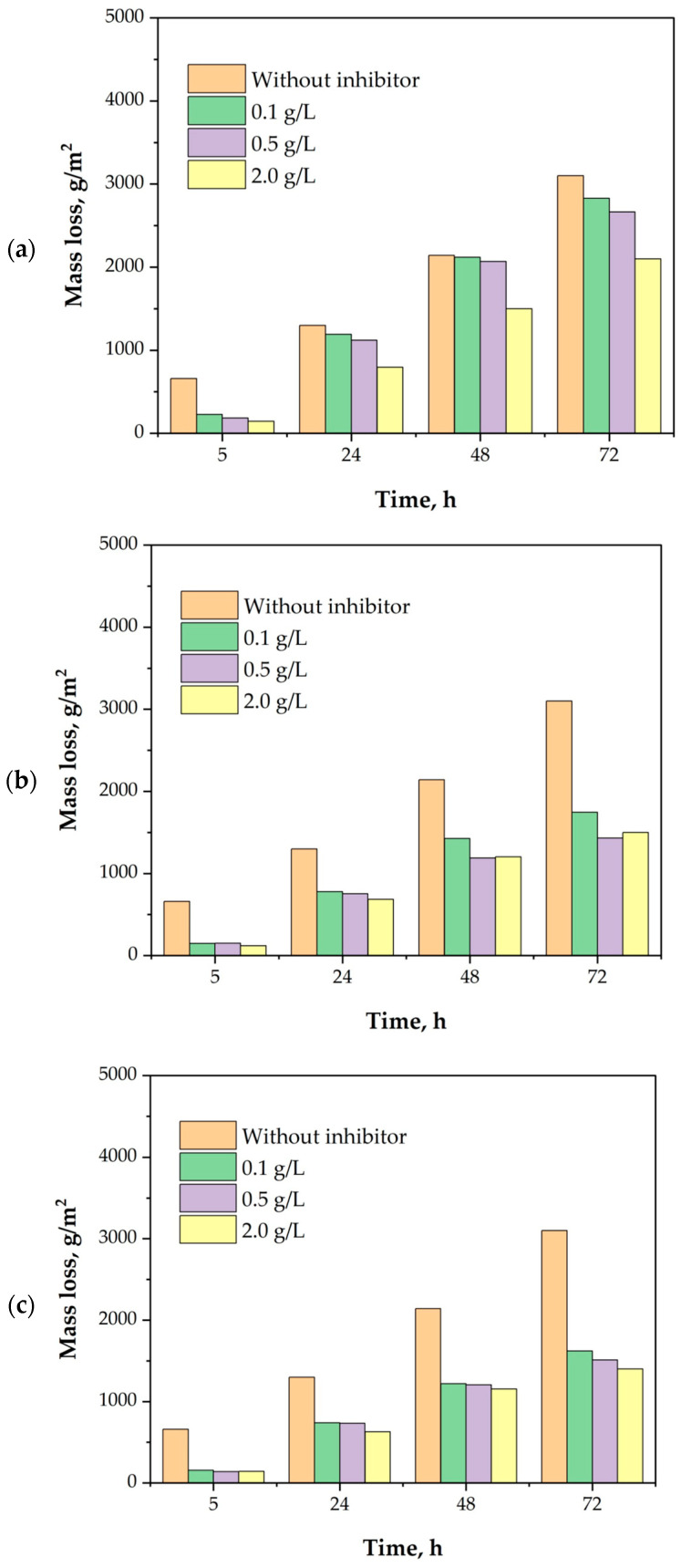
Effect of oxyphosphonate concentration on the mass loss of EP-450 steel in 10% FeCl_3_ at 20 to 23 °C (**a**) dimethyl(1-hydroxycyclohexyl)phosphonate; (**b**) dimethyl(4-hydroxytetrahydro-2H-thiopyran-4-yl)phosphonate; (**c**) dimethyl[1-(2-ethoxyethyl)-4-hydroxypiperidin-4-yl]phosphonate).

Mass-loss measurements show an almost linear increase with immersion time (Figure 4). The slope of dependence decreases with increasing inhibitor concentration, from approximately ~42 g/m^2^·h in the uninhibited solution to ~29 g/m^2^·h at 2.0 g/L, indicating a reduction in corrosion kinetics. This behavior reflects the continuous propagation of pits once the passive layer collapses. After 72 h, the corrosion rate of EP-450 reaches ~49 mm/year, which is characteristic of severe localized attack under ASTM G48-type conditions. Similar high rates have been reported for ferritic–martensitic steels in strong chloride oxidizers, especially when carbides and microstructural heterogeneities act as pit-nucleation sites [22,23].

Overall, the results confirm that EP-450 steel exhibits extremely poor resistance to pitting corrosion in ferric chloride media. The rapid initiation within 5 h and aggressive propagation over 72 h underline the critical role of microstructural features, particularly carbide precipitation, in controlling localized corrosion behavior. These findings highlight the necessity of applying effective organic inhibitors to suppress the rapid breakdown of passivity and to stabilize surface films under strong chloride attack.

### 2.2. Corrosion Behavior of EP-450 Steel in the Presence of Inhibitor

**Figure 5:**
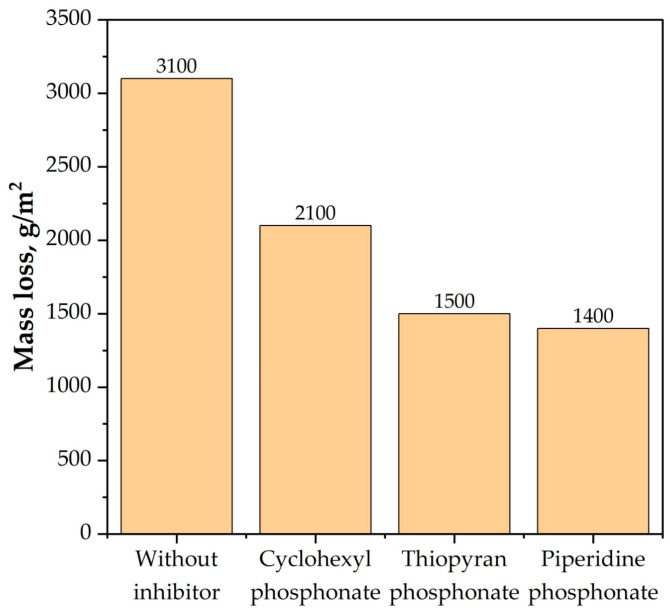
Mass loss of EP-450 steel in an FeCl_3_·6H_2_O solution in the presence of various oxyphosphonates (2.0 g/L, 72 h).

The addition of dimethyl(1-hydroxycyclohexyl)phosphonate to the chloride solution reduced the corrosion activity of EP-450 steel. The average corrosion rates in Table 1 decrease with increasing inhibitor concentration. The change from 49 mm/year in the uninhibited solution to 33 mm/year at 2.0 g/L corresponds to a reduction in metal loss of about 33% and a protection coefficient Z of 32%. Organic phosphonates are generally understood to inhibit corrosion through the adsorption of inhibitor molecules on the steel surface and the formation of a compact protective interfacial layer. Phosphonate groups may interact with iron through coordination and hydrogen bonding, which can enhance interfacial film stability and reduce dissolution processes [24]. The adsorbed layer likely restricts access of oxygen and chloride ions to active sites and decreases the rate of anodic dissolution. At a concentration of 2.0 g/L, the inhibitor exhibits the highest protective efficiency, consistent with increased surface coverage (Table 1). All reported values correspond to the average of three independent measurements and are consistent with the data presented in Figure 5. At lower concentrations in the range 0.125 to 0.5 g/L, surface coverage remains partial, and the protective effect is weak.

Figure 4 illustrates the time-dependent mass-loss profiles at 5, 24, 48, and 72 h for dimethyl(1-hydroxycyclohexyl)phosphonate (a), dimethyl(4-hydroxytetrahydro-2H-thiopyran-4-yl)phosphonate (b), dimethyl[1-(2-ethoxyethyl)-4-hydroxypiperidin-4-yl]phosphonate) (c). These data confirm that all inhibitors exhibit a similar time-dependent trend, with reduced mass loss at higher concentrations, while differing in overall inhibition efficiency.

At 0.1 g/L, the level of protection is limited, which indicates incomplete surface coverage. A marked improvement appears at 0.5 g/L, and the highest suppression of mass loss is recorded at 2.0 g/L. The results align with recent observations for organophosphorus inhibitors on ferritic and martensitic steels, where concentration-dependent adsorption leads to gradual blocking of active areas [25]. The time-dependent reduction in mass loss may also suggest progressive consolidation of the adsorbed layer and reduced susceptibility of the passive film to localized breakdown during longer immersion [26].

The combined evidence identifies 2.0 g/L as the most effective tested concentration for improving surface protection and minimizing mass loss under the present conditions.

### 2.3. Comparison of the Three Inhibitors

Follow-up corrosion tests demonstrated that the three oxyphosphonates exhibit markedly different inhibition efficiencies depending on the nature of the heteroatom incorporated into the molecule. Figure 5 shows the mass-loss data for EP-450 steel exposed to FeCl_3_·6H_2_O solution in the presence of each inhibitor under identical conditions (2.0 g/L, 72 h). For graphical clarity, the mass-loss values in Figure 5 are presented rounded to two significant figures. To provide a clearer comparison of the heteroatom effect under identical exposure conditions, the main corrosion parameters for the uninhibited system and for the three oxyphosphonate inhibitors at 2.0 g/L after 72 h are summarized in Table 2.

Among the three compounds, dimethyl[1-(2-ethoxyethyl)-4-hydroxypiperidin-4-yl]phosphonate (piperidine phosphonate) showed the highest inhibition performance. The corrosion rate decreased by more than 55% relative to the uninhibited control, yielding a protection efficiency of approximately Z ≈ 57% (Equation (3)).

The sulfur-containing derivative, dimethyl(4-hydroxytetrahydro-2H-thiopyran-4-yl)phosphonate (thiopyran phosphonate), exhibited a moderate protective effect, corresponding to a 52% reduction in corrosion rate, compared to the nitrogen-containing derivative.

As illustrated in Figure 5, the comparative inhibition performance appears to depend, at least in part, on how heteroatom substitution may influence adsorption configuration and interfacial packing at the EP-450 surface. The cyclohexyl-based phosphonate provided the weakest protection, whereas incorporation of S and especially N into the heterocycle improved inhibition, consistent with alternative adsorption orientations and differences in film compactness. Because direct spectroscopic identification of bonding/adsorption configurations was not performed here, the role of N or S is discussed as a plausible secondary coordination/anchoring contribution that can affect effective surface coverage and defect density rather than as conjugation-driven activation of the P=O group. Accordingly, the protection efficiency increases in the sequence cyclohexylphosphonate < thiopyranphosphonate < piperidinephosphonate. This sequence reflects the experimentally observed inhibition performance and should not be interpreted as a direct thermodynamic ranking of adsorption strength, since adsorption-isotherm parameters were calculated only for the cyclohexylphosphonate system. Combined with the comparative inhibition data, these results suggest that heteroatom substitution, particularly N and then S, may be associated with more favorable adsorption configurations and improved interfacial packing on Fe/Cr surface sites, which can enhance barrier stability against chloride-induced breakdown.

The strongest inhibiting effect was obtained for the phosphonate containing a nitrogen atom in the ring, which is consistent with the high adsorption propensity of N-containing inhibitors. In the absence of direct spectroscopic confirmation, the N contribution is interpreted as a plausible additional adsorption interaction that can favor higher effective surface coverage and improved film compactness, thereby supporting passivity under oxidizing chloride conditions [27,28]. The nitrogen-containing inhibitor produced the highest inhibition, while the sulfur-containing derivative showed an intermediate effect under identical conditions. In the absence of direct surface-chemical identification in the present work, the influence of N or S is discussed as a plausible secondary anchoring contribution (in addition to phosphonate oxygen binding) that can modify adsorption orientation, surface packing, and defect density within the protective layer, rather than as a conjugation-driven activation of the P=O group. The sulfur-containing compound exhibited an intermediate inhibiting effect, which may reflect heteroatom-dependent adsorption configurations that produce less compact or more defect-rich protective films under oxidizing chloride conditions. However, distinguishing between packing effects and specific bonding interactions requires surface-sensitive analysis. The observed inhibition trend appears to correlate well with the donor properties of the heteroatom, apparent adsorption strength, and its potential to stabilize the interfacial protective film.

The superior performance of the N-containing oxyphosphonate may be consistent with the formation of a more compact protective layer under the present conditions. A plausible interpretation is that, alongside phosphonate oxygen anchoring, the ring nitrogen may provide an additional adsorption interaction that favors higher effective surface coverage and reduced defect density; however, confirming the specific bonding configuration requires surface-sensitive characterization. Similar behavior has been widely documented for N-containing organic inhibitors, where stronger coordination leads to greater surface coverage and improved corrosion resistance [29,30,31].

Overall, our results (Figure 4 and Figure 5, Table 2 and Table 3) indicate that replacing hydroxyalkyl fragments with N- or S-containing heterocycles substantially improves the inhibitory action of phosphonates under the tested conditions. Among the tested heteroatoms, nitrogen provides the most pronounced reduction in pitting susceptibility, which may be associated with its donor properties and possible additional interactions consistent with its higher electron density and stronger coordination ability toward iron- and chromium-rich passive layers. The observed inhibition trend correlates well with the donor properties of the heteroatom, adsorption strength, and its capacity to stabilize the interfacial protective film.

The present results agree with previous reports describing enhanced inhibition efficiency of phosphorus and nitrogen-containing species when multiple functional groups act cooperatively [32,33]. Studies by Tolstolutskaya et al. [2] also show that steel microstructure and carbide precipitates exert a decisive influence on corrosion mechanisms and inhibitor adsorption. Taken together, the inhibition effect observed for the present compounds appears to be multifactorial and likely reflects both the chemical nature of the heteroatom and the specific microstructural features of EP-450 steel, which promote pit initiation.

Because the present study is based primarily on gravimetric kinetics and post-exposure optical microscopy, without XPS, SEM-EDS surface mapping, EIS, or potentiodynamic polarization measurements, the specific bonding/adsorption configurations of each oxyphosphonate on EP-450 cannot be uniquely assigned (Figure 4 and Figure 5, Table 2 and Table 3). Nevertheless, the data are consistent with heteroatom-dependent adsorption configurations in which phosphonate oxygen atoms provide primary anchoring, while N or S may contribute secondary interactions that influence molecular orientation, packing density, and the probability of defect formation within the protective film. Verification of these adsorption configurations will require surface-sensitive characterization (e.g., XPS and/or in situ EIS) in future work. For N-containing oxyphosphonates, both a cooperative (O + N) binding adsorption configuration and a competing-orientation (alternating anchoring) adsorption configuration are plausible, but the present dataset cannot discriminate between these regimes. These findings further suggest that the chemical structure of an inhibitor is important, even when it does not directly influence the chemical activity or accessibility of the main active group, since it can still affect adsorption geometry and interfacial packing. From a molecular perspective, the observed inhibition trend (cyclohexyl < thiopyran < piperidinyl) can be rationalized by heteroatom-dependent adsorption geometry and surface packing rather than by changes in intrinsic phosphonate reactivity. Nitrogen and sulfur heteroatoms introduce additional lone-pair donor sites that can plausibly participate in secondary interactions with Fe- and Cr-containing surface sites, influencing molecular orientation and effective surface coverage. The higher electronegativity and directional lone-pair availability of nitrogen may be consistent with more compact interfacial packing and reduced defect density within the adsorbed layer. These considerations provide a qualitative structure–activity interpretation that complements the gravimetric data without implying a uniquely defined bonding motif. Although the inhibition efficiencies obtained here are moderate compared with optimized industrial corrosion inhibitors, the results are useful for identifying structure-dependent trends under severe FeCl_3_-induced pitting conditions.

### 2.4. Adsorption Behavior of Dimethyl(1-Hydroxycyclohexyl)phosphonate

**Figure 6:**
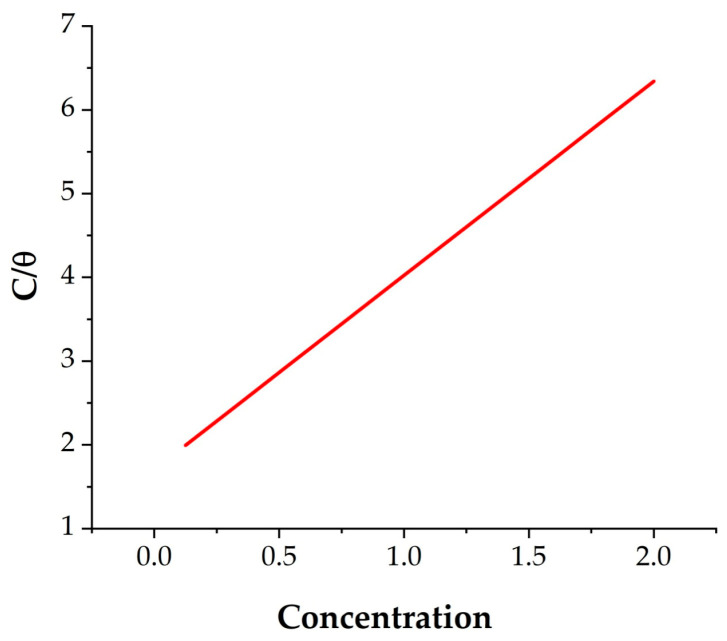
Plot of *C*/*θ* versus *C* for dimethyl(1-hydroxycyclohexyl)phosphonate on EP-450 at 72 h.

**Figure 7:**
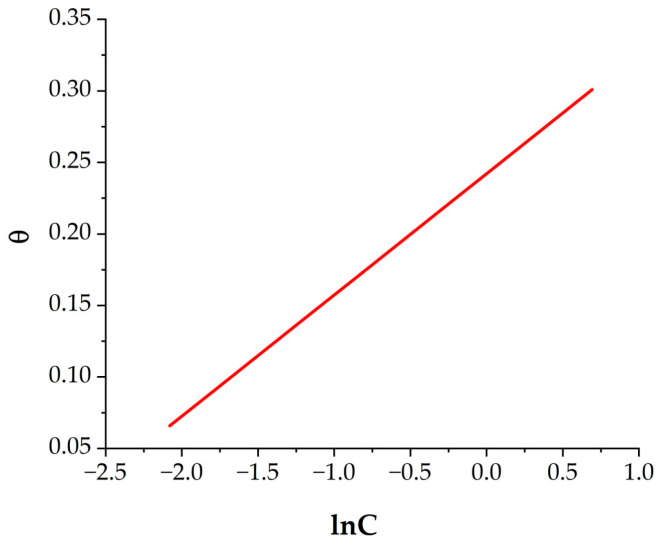
Plot of *θ* versus ln*C* for dimethyl(1-hydroxycyclohexyl)phosphonate on EP-450 at 72 h.

The adsorption analysis was performed using corrosion rate data from gravimetric measurements (Table 1). The degree of surface coverage (*θ*) was calculated according to Equation (4) (Section 3.7), and the resulting values are summarized in Table 3. These data were subsequently used to construct adsorption isotherms, including the Langmuir plot (*C*/*θ* versus *C*, Figure 6) and the Temkin plot (*θ* versus ln*C*, Figure 7). The adsorption equilibrium constant (*K_ads_*) was determined from the intercept of the Langmuir plot, and the standard free energy of adsorption (Δ*G°_ads_*) was calculated using Equation (7). Accordingly, the calculated surface coverage (*θ*) should be regarded as an apparent parameter derived from corrosion-rate reduction, rather than a direct measure of true microscopic surface occupation.

It should be noted that the adsorption analysis was performed only for dimethyl(1-hydroxycyclohexyl)phosphonate. Therefore, the calculated *K_ads_* and Δ*G°_ads_* values are not used for direct thermodynamic comparison among all three oxyphosphonate inhibitors. The inhibition sequence for the N- and S-containing compounds is discussed on the basis of measured corrosion-rate reduction under identical exposure conditions.

The adsorption process was evaluated using the Langmuir and Temkin models. The Langmuir plot produced a linear relationship with an adsorption constant *K_ads_* of 1.14 L/g. The adsorption behavior was evaluated using the Langmuir isotherm model. The linear dependence of *C*/*θ* versus *C* (Figure 6) suggests that adsorption of dimethyl(1-hydroxycyclohexyl)phosphonate can be approximated by a Langmuir-type model, consistent with apparent monolayer-like behavior on the EP-450 surface. The calculated *θ* values were used to construct adsorption isotherms based on the equations introduced in Section 3.7, enabling evaluation of inhibitor–surface interactions. The corresponding standard free energy of adsorption was −24.8 kJ/mol. This value indicates mixed physisorption and chemisorption. In corrosion-inhibition studies, Δ*G°_ads_* values around −20 kJ/mol or less negative are commonly associated with predominantly physical adsorption, whereas values close to or more negative than −40 kJ/mol are usually attributed to chemisorption involving stronger charge sharing or coordination. The value obtained here, −24.8 kJ/mol, lies in the intermediate region; therefore, adsorption of dimethyl(1-hydroxycyclohexyl)phosphonate on EP-450 is interpreted as mixed physisorption–chemisorption, with the adsorption parameters considered as apparent values derived from gravimetric corrosion-rate reduction. The Temkin model produced an interaction parameter f of approximately 4.1 and a lower *K_ads_* of approximately 0.52 L/g (Equation (6)), which may reflect repulsive interactions between adsorbed molecules and limited surface packing. The calculated surface coverage increased from 0.06 at 0.125 g/L to 0.33 at 2.0 g/L. These results suggest relatively weak apparent adherence of cyclohexyl phosphonate to the steel surface. Limited electron donation from this structure may restrict the formation of a more stable chemisorbed layer. Reports on phosphonate and heteroatom-based inhibitors support this interpretation and show that adsorption strength increases with the presence of donor atoms such as nitrogen and sulfur in the inhibitor structure [31,34].

The surface coverage (*θ*) values listed in Table 3 were calculated according to Equation (4) (Section 3.7), using gravimetric mass-loss data obtained in the absence and presence of inhibitor. The adsorption parameters derived from corrosion-rate data (Table 3, based on values reported in Table 1 and Figure 4) should be interpreted as effective (apparent) values under dynamic exposure, where adsorption/desorption and film reorganization may occur concurrently with pit nucleation processes. Accordingly, the protective layer is discussed as an interfacial state that may evolve toward greater compactness with time and concentration, rather than as a permanently fixed monolayer. Electrochemical techniques such as potentiodynamic polarization or EIS were not employed in this study because the focus was on accelerated pitting severity under strongly oxidizing ferric-chloride conditions, where gravimetric mass-loss measurements provide a more representative assessment of localized damage evolution. Under such conditions, electrochemical parameters may be dominated by rapid passive-film breakdown and pit propagation rather than by steady-state interfacial kinetics.

## 3. Materials and Methods

### 3.1. Materials

The material studied was a ferritic-martensitic stainless steel of grade Cr13Mo2NbVB (EP-450), which is widely used as a structural alloy in advanced nuclear systems, particularly in liquid-metal-cooled fast reactors, due to its favorable radiation tolerance and mechanical stability [1,35]. This steel exhibits high mechanical strength, excellent radiation resistance, and good tolerance to thermal stresses. However, it remains susceptible to localized degradation, particularly pitting corrosion, when exposed to chloride-containing environments.

The chemical composition of the steel was determined by energy-dispersive X-ray spectroscopy (EDS) using an X-Max detector (80 mm^2^) (Oxford Instruments, Abingdon, UK) mounted on a JEM-2100 transmission electron microscope (JEOL, Tokyo, Japan). The average elemental composition (wt.%) was as follows: Si—0.3; Mn—0.5; Cr—12.8; Mo—1.0; Ni—0.2; V—0.2; Nb—0.1; Fe—balance.

Carbon, phosphorus, and sulfur contents were not quantified due to the limitations of EDS for detecting light elements with sufficient accuracy [1].

### 3.2. Synthesis of Oxyphosphonates

The oxyphosphonates were synthesized under standard Abramov reaction conditions, following the general protocol described by Kystaubayeva et al. [36]. Comprehensive procedures for the preparation, structural characterization, and spectral identification of dimethyl(1-hydroxycyclohexyl)phosphonate and dimethyl(1-(2-ethoxyethyl)-4-hydroxypiperidin-4-yl)phosphonate are reported in Askar et al. [37] and in the work of Kystaubayeva et al., which also details the complexation behavior of these ligands with Pd(II) and Pt(II) ions and the catalytic performance of the Pd(II) complex in Suzuki–Miyaura cross-coupling reactions. The synthesis of dimethyl(4-hydroxy-2,2-dimethyltetrahydro-2H-thiopyran-4-yl)phosphonate was likewise conducted under Abramov reaction conditions. Prior to filtration, the reaction mixture was stored at a low temperature to induce precipitation. The resulting solid was isolated by filtration and purified by recrystallization from hexane, consistent with the procedures used for the other oxyphosphonates. The final product was obtained in 37% yield (m.p. 101–102 °C). All IR, ^1^H NMR, ^13^C NMR, COSY, HMQC, and HMBC spectra obtained for this compound are provided in the Supporting Information (Figures S1–S6).

IR (KBr, ν, cm^−1^): 3308, 2850–3005, 1369–1461, 1237, 1053–1069 (Figure S1);

^1^H NMR (CDCl_3_, δ, ppm): 1.17 (s, 3H, C–CH_3_), 1.52 (s, 3H, C–CH_3_), 1.79–3.16 (m, 6H, -CH_2_-), 3.71–3.75 (m, 6H, O–CH_3_), 4.79 (s, 1H, O–H) (Figure S2);

^13^C NMR (CDCl_3_, δ, ppm): 20.29–20.44 (C-2), 29.80 (C-12), 32.17 (C-3), 33.15 (C-11), 38.89–39.05 (C-6), 43.97 (C-5), 53.64–54.06 (C-9, C-14), 71.63–73.27 (C-4) (Figure S3).

### 3.3. Sample Preparation

Flat specimens with minimum dimensions of 10 × 10 × 2 mm were prepared by cutting sections from the walls of unirradiated cladding tubes of fuel assemblies. The thickness of the samples corresponded to the actual thickness of reactor components. The surfaces were sequentially ground and polished to a mirror finish using abrasive papers and diamond pastes of gradually decreasing grit size. The final surface roughness after polishing was Ra < 0.05 μm, ensuring uniformity and minimizing the influence of surface defects on corrosion initiation.

Prior to testing, the specimens were rinsed with distilled water, degreased with ethanol, and dried under ambient laboratory conditions. The mass of each sample was measured on analytical balances KERN-770 (KERN and SOHN GmbH, Balingen, Germany) with an accuracy of 0.0001 g. Geometric dimensions were recorded using precision micrometers (Mitutoyo Co., Kanagawa, Japan) with an accuracy of ±0.01 mm.

### 3.4. Corrosive Medium

A 10% aqueous solution of ferric chloride (FeCl_3_·6H_2_O) was selected as the corrosive medium due to its widespread application in standardized accelerated pitting corrosion tests, particularly ASTM G48 Method C, which is often used for evaluating the localized corrosion resistance of stainless steels. The solution was prepared by dissolving 100 g of FeCl_3_·6H_2_O in 900 mL of distilled water, adjusted to a density of 1.049 g/cm^3^, and aged for 24 h in a sealed container at room temperature. Unless specified otherwise, all corrosion exposure experiments were conducted at 25 ± 1 °C, ensuring reproducibility and comparability with established testing protocols.

### 3.5. Tested Inhibitors

Three oxyphosphonate compounds with different heteroatoms were studied as organic corrosion inhibitors:

1. Dimethyl(1-hydroxycyclohexyl)phosphonate (cyclohexylphosphonate).
2. Dimethyl[1-(2-ethoxyethyl)-4-hydroxypiperidin-4-yl]phosphonate (piperidinylphosphonate, containing a nitrogen (N) heteroatom in the ring.
3. Dimethyl(4-hydroxytetrahydro-2H-thiopyran-4-yl)phosphonate (thiopyranphosphonate), incorporating a sulfur (S) atom in the heterocycle.

All inhibitors were introduced into the corrosive medium at concentrations of 0.125, 0.5, and 2.0 g/L. Each compound was synthesized in our laboratory and purified to ≥98%, with structural confirmation performed via NMR and IR spectroscopy (details provided in the Supporting Information). This ensured that the observed corrosion protection effects were directly attributable to the molecular structure and heteroatom nature of the inhibitors.

### 3.6. Corrosion Testing Procedure

Experiments were performed in 100 mL glass flasks in which the specimens were fully immersed. Each specimen was immersed in a separate glass flask under fully immersed conditions, ensuring that all surfaces of the sample were uniformly exposed to the corrosive solution without shielding or contact with the container walls. To ensure experimental accuracy, three samples were tested simultaneously under identical conditions. All reported corrosion-rate values represent the average of three independent measurements, and the observed trends were reproducible within experimental uncertainty. Exposure durations were 5, 24, 48, and 72 h. After each interval, the samples were removed, rinsed with running water and distilled water, degreased with ethanol, ultrasonically cleaned, dried, and reweighed. Mass losses were used to calculate corrosion rates.

### 3.7. Calculation of Corrosion Rate

The corrosion rate (CR) was calculated using the following expression:

(1) $$P=\frac{8.76K_{m}}{\rho },mm/year$$

The conversion factor $K_{m}$ was determined using the following expression:

(2) $$K_{m}=\frac{\Delta m}{S\tau },g/\left(m^{2}\times h\right)$$

where Δ*m*—mass loss (g), *S*—specimen surface area (m^2^), *τ*—exposure time (h), ρ—density of steel (g/cm^3^).

The inhibition efficiency was evaluated using the following expression:

(3) $$Z=100\times \frac{K_{m1}-K_{m2}}{K_{m1}},\%$$

where *K_m_*_1_ and *K_m_*_2_ are the corrosion rates in the absence and presence of inhibitor, respectively.

Adsorption isotherm calculations: to assess the adsorption behavior of oxyphosphonate inhibitors on EP-450 steel, the degree of surface coverage (*θ*) was calculated from corrosion-rate data using

(4) $$\theta =\frac{K_{m1}-K_{m2}}{K_{m1}}$$

The obtained *θ* values were used to evaluate adsorption according to the Langmuir and Temkin isotherm models, which are widely applied in corrosion inhibition studies to describe inhibitor–metal surface interactions [38,39].

Adsorption according to the Langmuir model was examined using the linearized form:

(5) $$\frac{C}{\theta }=\frac{1}{K_{ads}}+C$$

where *C* is the inhibitor concentration, and *K_ads_* is the adsorption equilibrium constant (Figure 6). These equations were used to calculate surface coverage and to model adsorption behavior, as discussed in Section 2.4.

From the linear regression of the Langmuir plot (*C*/*θ* versus *C*) shown in Figure 6, which exhibits good linearity, the relationship was expressed as y = ax + b, where the intercept corresponds to 1/*K_ads_*. Therefore, the adsorption equilibrium constant was calculated as

(6) $$K_{ads}=\frac{1}{b}$$

The standard free energy of adsorption (Δ*G°_ads_*) was calculated using the thermodynamic relation based on the adsorption equilibrium constant, which is commonly derived from the Langmuir isotherm and reflects the Gibbs free energy change associated with inhibitor adsorption [38,39]:

(7) $$\Delta G_{ads}^{\circ }=-RTln(55.5K_{ads})$$

where 55.5 is the molar concentration of water (mol/L), T is the absolute temperature (K), corresponding to 298 K (25 °C), and R is the universal gas constant (8.314 J/mol·K). Based on the calculated negative values of Δ*G°_ads_*, inhibitor adsorption appears thermodynamically favorable under the tested conditions. Thus, Δ*G°_ads_* provides a thermodynamic interpretation of the observed reduction in corrosion rate and may be used to discuss the apparent adsorption strength in relation to inhibition efficiency.

The Temkin isotherm was applied to evaluate surface interactions and heterogeneity according to:

(8) $$\theta =\frac{1}{f}(lnK_{ads}+lnC)$$

where *f* is the Temkin interaction parameter, and *K_ads_* is the Temkin adsorption constant. For the linear form of the Temkin isotherm, $\theta =(1/f)(ln\;K_{ads}+ln\;C)$, the slope of the plot of θ versus lnC  is equal to 1/f. Accordingly, the interaction parameter f was determined as the reciprocal of the slope [39].

Parameters *f* and *K_ads_* were obtained from the linear dependence of *θ* on ln*C* (Figure 7).

Surface morphology was examined using optical microscopy in reflected light mode at ×200 magnification. Optical images were used to confirm the localized character of corrosion qualitatively. No statistical image analysis of pit depth, pit density, or pit area fraction was performed in the present work.

Surface-sensitive techniques (e.g., XPS, EIS) were not employed in this study; therefore, adsorption parameters are interpreted as effective values derived from macroscopic corrosion data.

It should be emphasized that gravimetric measurements quantify total material loss and do not resolve pit density or depth; therefore, the results are interpreted as indicators of overall pitting severity rather than detailed pit morphology.

Electrochemical techniques such as EIS and potentiodynamic polarization were not applied in this study because the strongly oxidizing FeCl_3_ medium promotes rapid passive-film breakdown and unstable electrochemical responses, making gravimetric assessment more representative of cumulative pitting damage under ASTM G48-type conditions.

## 4. Conclusions

The present study establishes an empirical structure–activity relationship for three oxyphosphonate inhibitors differing in heteroatom composition under severe FeCl_3_-induced pitting conditions of ferritic–martensitic EP-450 steel. The inhibition efficiency increased in the sequence cyclohexylphosphonate < thiopyranphosphonate < piperidinylphosphonate, indicating that the incorporation of heteroatoms, particularly nitrogen, can improve the protective performance of oxyphosphonate molecules.

The investigated oxyphosphonates reduced corrosion of EP-450 steel in ferric chloride solution, as evidenced by decreased mass loss and improved inhibition efficiency. For the cyclohexylphosphonate system, the adsorption behavior was reasonably approximated by a Langmuir-type model, consistent with the formation of an apparent protective surface layer that may limit metal dissolution. The superior performance of the piperidinylphosphonate may be associated with the presence of nitrogen-containing functionality, which can plausibly affect adsorption orientation, interfacial packing, and effective surface coverage. At the same time, gravimetric measurements provide only a macroscopic assessment of corrosion severity and should be interpreted as reflecting overall material degradation rather than detailed pit morphology. Accordingly, the calculated adsorption should be regarded as apparent values derived from corrosion-rate reduction. Therefore, the proposed heteroatom-dependent adsorption mechanism should be considered as a plausible interpretation of the observed inhibition trend rather than as direct confirmation of a specific bonding mode.

Future investigations should include electrochemical impedance spectroscopy, potentiodynamic polarization, SEM-EDS or XPS surface analysis, SEM-based pit density evaluation, and profilometry-based pit depth measurements to verify the proposed adsorption mechanism and quantify localized corrosion damage more precisely.

## Figures and Tables

**Table 1:** Average corrosion rate of EP-450 steel at different concentrations of dimethyl(1-hydroxycyclohexyl)phosphonate.

Inhibitor Concentration (g/L)	Corrosion Rate P (mm/Year)
0	49
0.125	46
0.5	44
2.0	33

**Table 2:** Comparative corrosion parameters of EP-450 steel in 10% FeCl_3_ solution in the absence and presence of oxyphosphonate inhibitors at 2.0 g/L after 72 h.

System	Main Structural Motif	Mass Loss, g/m^2^	Corrosion Rate, mm/Year	Surface Coverage, *θ*	Inhibition Efficiency, %
Without inhibitor	–	3100	49	–	
Cyclohexylphosphonate	C-containing cyclic fragment	2100	33	0.327	33
Thiopyranphosphonate	S-containing heterocycle	1500	24	0.510	52
Piperidinylphosphonate	N-containing heterocycle	1400	22	0.551	55

**Table 3:** Calculation of surface coverage (*θ*) for dimethyl(1-hydroxycyclohexyl)phosphonate.

*C* (g/L)	*P* (mm/Year)	*θ* = (49 − *P*)/49	*C*/*θ*
0.125	46	0.0612	2.04
0.5	44	0.1020	4.90
2.0	33	0.3265	6.12

## Data Availability

The datasets used and/or analyzed during the present study are available from the corresponding author on reasonable request.

## Supplementary Materials

The following supporting information can be downloaded at: https://www.mdpi.com/article/10.3390/molecules31142504/s1. Figure S1: IR (KBr, ν, cm^−1^) spectrum of dimethyl(4-hydroxy-2,2-dimethyltetrahydro-2H-thiopyran-4-yl)phosphonate; Figure S2: ^1^H NMR (399.78 MHz, CDCl_3_) spectrum of dimethyl(4-hydroxy-2,2-dimethyltetrahydro-2H-thiopyran-4-yl)phosphonate; Figure S3: ^13^C NMR (100.53 MHz, CDCl_3_) spectrum of dimethyl(4-hydroxy-2,2-dimethyltetrahydro-2H-thiopyran-4-yl)phosphonate; Figure S4: ^1^H-^1^H COSY spectrum of dimethyl(4-hydroxy-2,2-dimethyltetrahydro-2H-thiopyran-4-yl)phosphonate; Figure S5: ^1^H-^13^C HMQC spectrum of dimethyl(4-hydroxy-2,2-dimethyltetrahydro-2H-thiopyran-4-yl)phosphonate; Figure S6: ^1^H-^13^C HMBC spectrum of dimethyl(4-hydroxy-2,2-dimethyltetrahydro-2H-thiopyran-4-yl)phosphonate.
