# Supplementary material for: Structure–Activity Relationship of Oxyphosphonate Inhibitors: Role of Heteroatoms in Controlling Pitting Corrosion of Ferritic–Martensitic Steel EP-450
Source: Molecules. 2026 Jul 17;31(14):2504. doi: 10.3390/molecules31142504 (PMC13415323; doi:10.3390/molecules31142504)
Supplement: Supplementary file 1 [file molecules-31-02504-s001.zip › molecules-4383716-supplementary.pdf]

Supplementary information

The IR spectra were recorded on a Bruker Alpha-P ATR FTIR (diamond crystal) spectrometer using potassium bromide (KBr) pellets, operating range 400-4000 cm^{-1}.

The ^{1}H and ^{13}C NMR spectra of the samples were recorded using a JNM-ECA 400 (Jeol) spectrometer, operating at frequencies of 399.78 MHz for ^{1}H and 100.53 MHz for ^{13}C, in deuterated chloroform ($CDCl_3$)

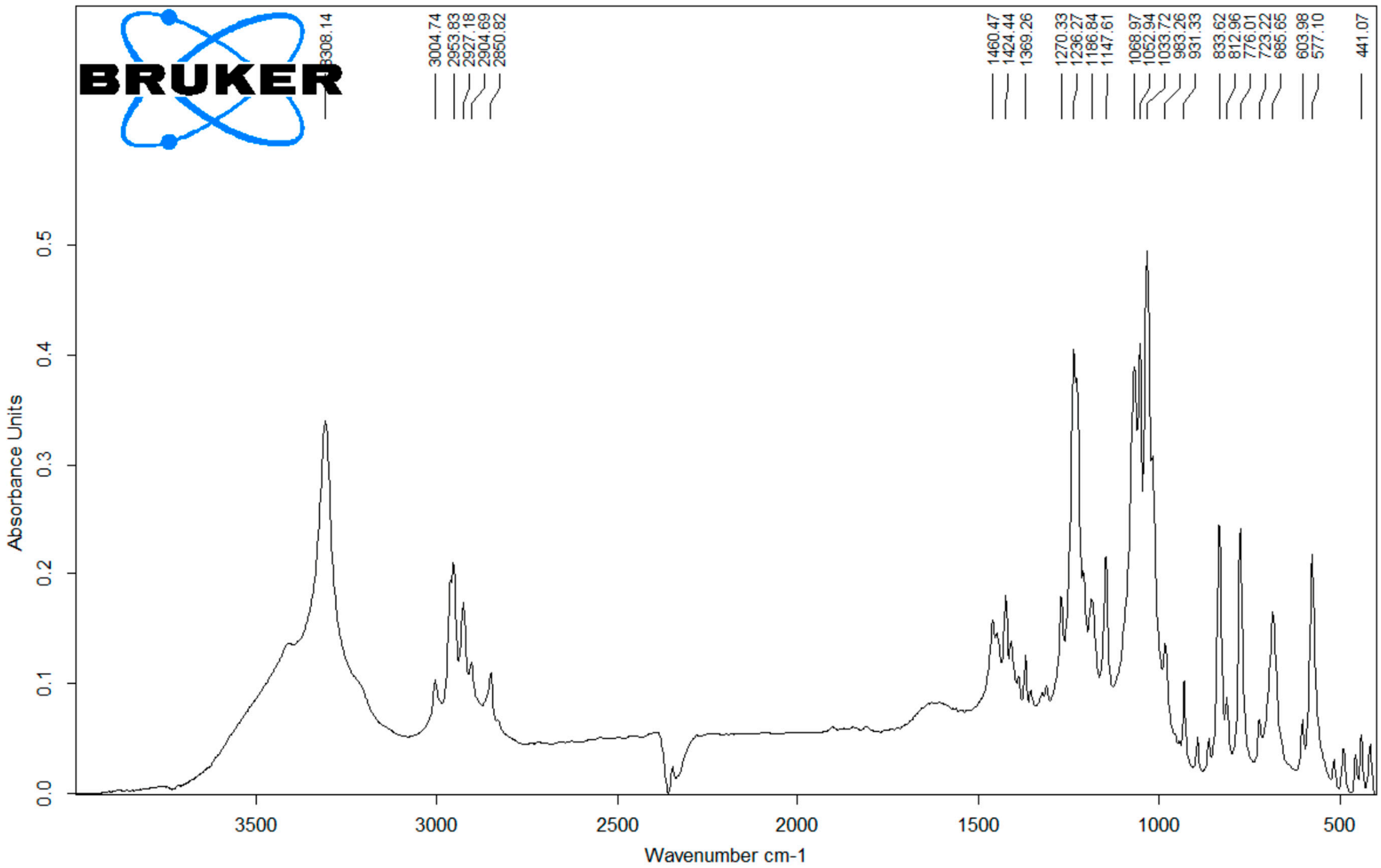

Figure S1. IR (KBr, ν, cm^{-1}) spectrum of dimethyl(4-hydroxy-2,2-dimethyltetrahydro-2H-thiopyran-4-yl)phosphonate.

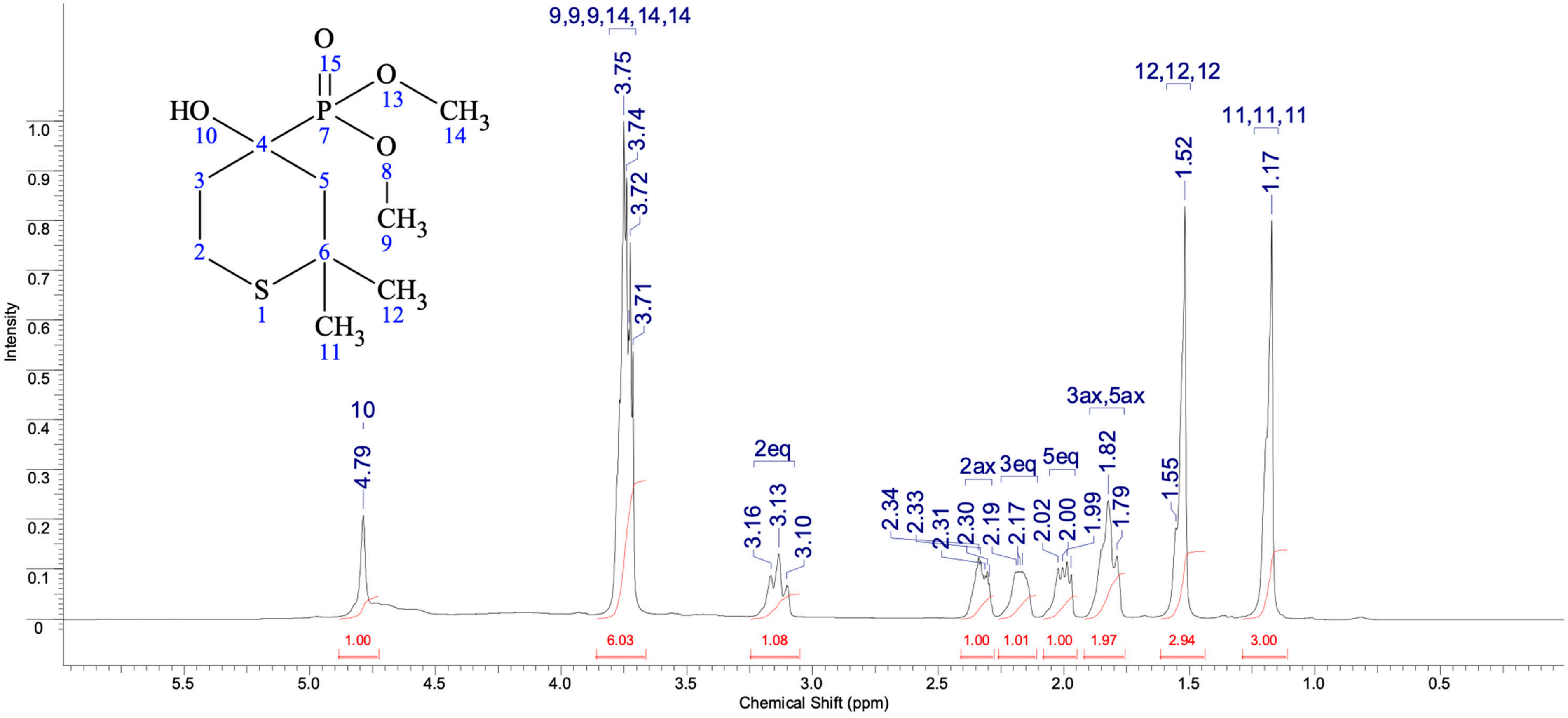

Figure S2. ^{1}H NMR (399.78 MHz, $CDCl_3$) spectrum of dimethyl(4-hydroxy-2,2-dimethyltetrahydro-2H-thiopyran-4-yl)phosphonate.

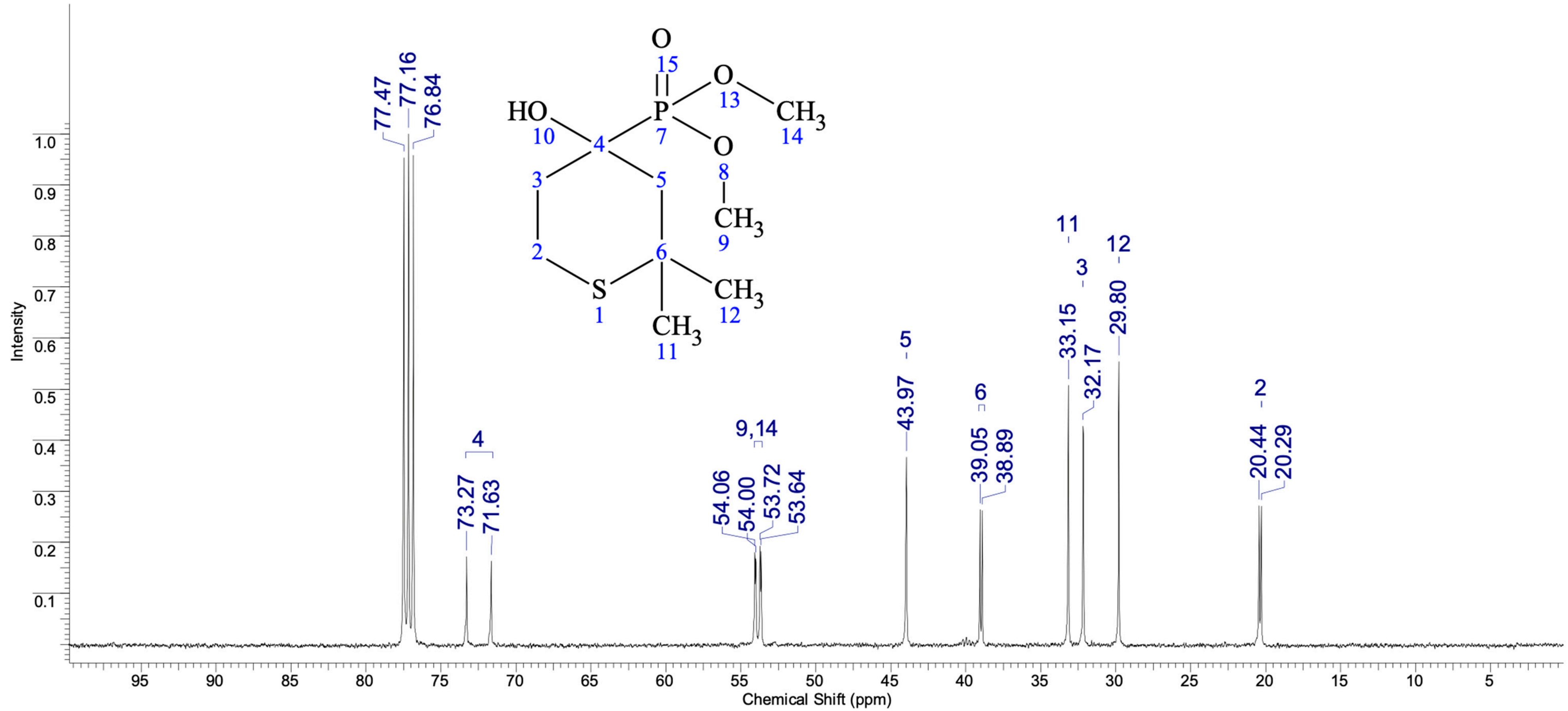

Figure S3. ^{13}C NMR (100.53 MHz, $CDCl_3$) spectrum of dimethyl(4-hydroxy-2,2-dimethyltetrahydro-2H-thiopyran-4-yl)phosphonate.

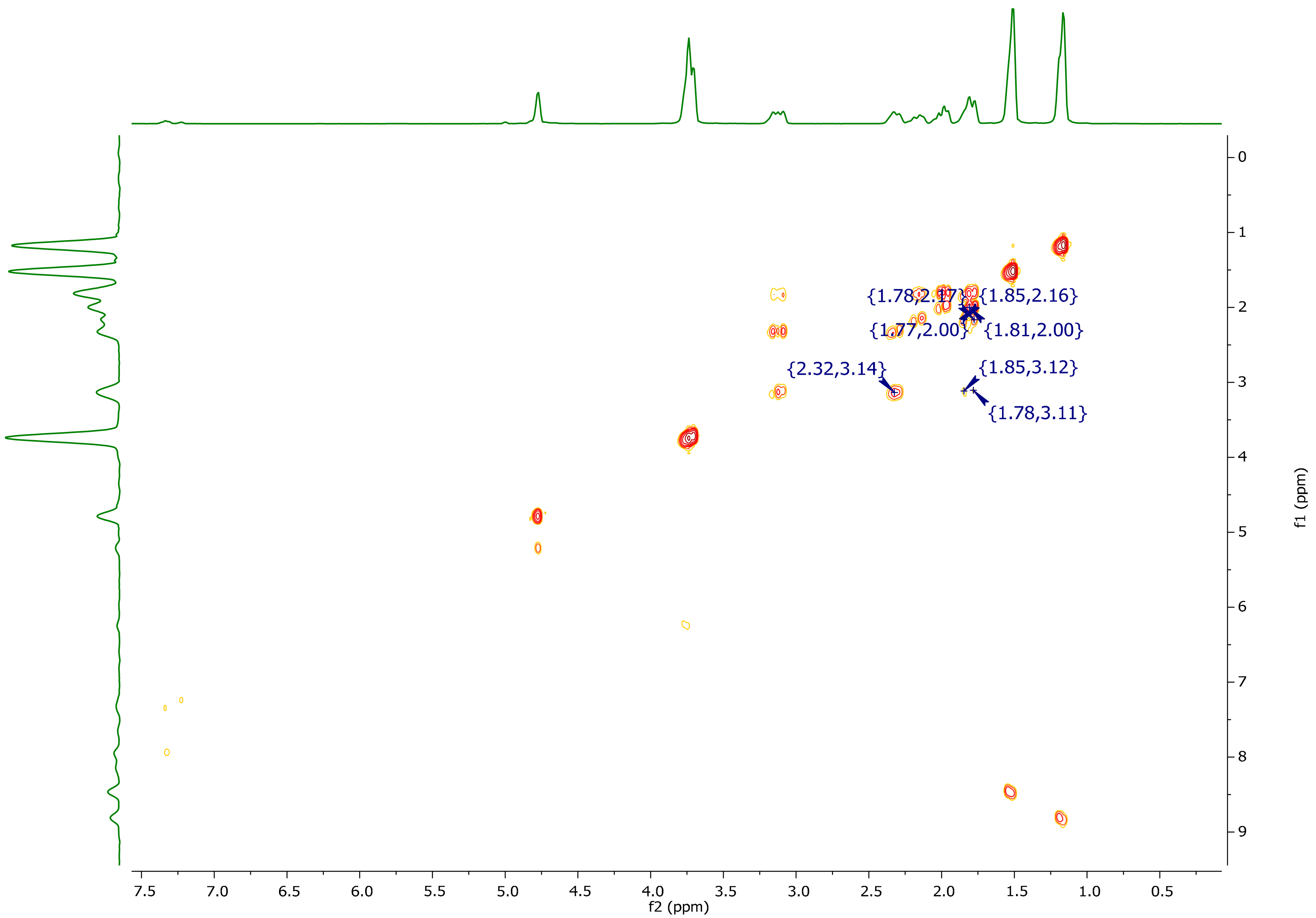

Figure S4. ^{1}H-^{1}H COSY spectrum of dimethyl(4-hydroxy-2,2-dimethyltetrahydro-2H-thiopyran-4-yl)phosphonate.

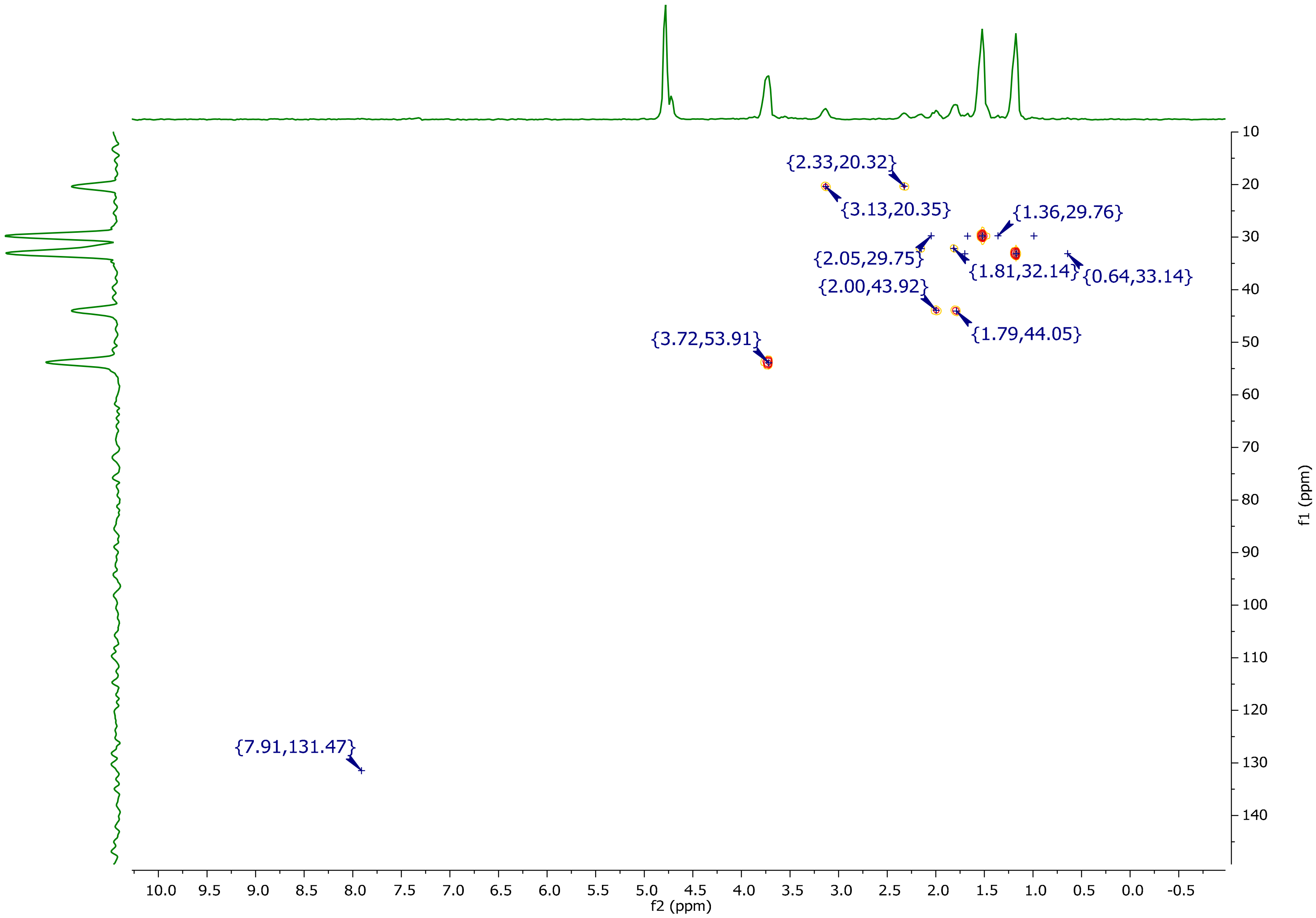

Figure S5. ^{1}H-^{13}C HMQC spectrum of dimethyl(4-hydroxy-2,2-dimethyltetrahydro-2H-thiopyran-4-yl)phosphonate.

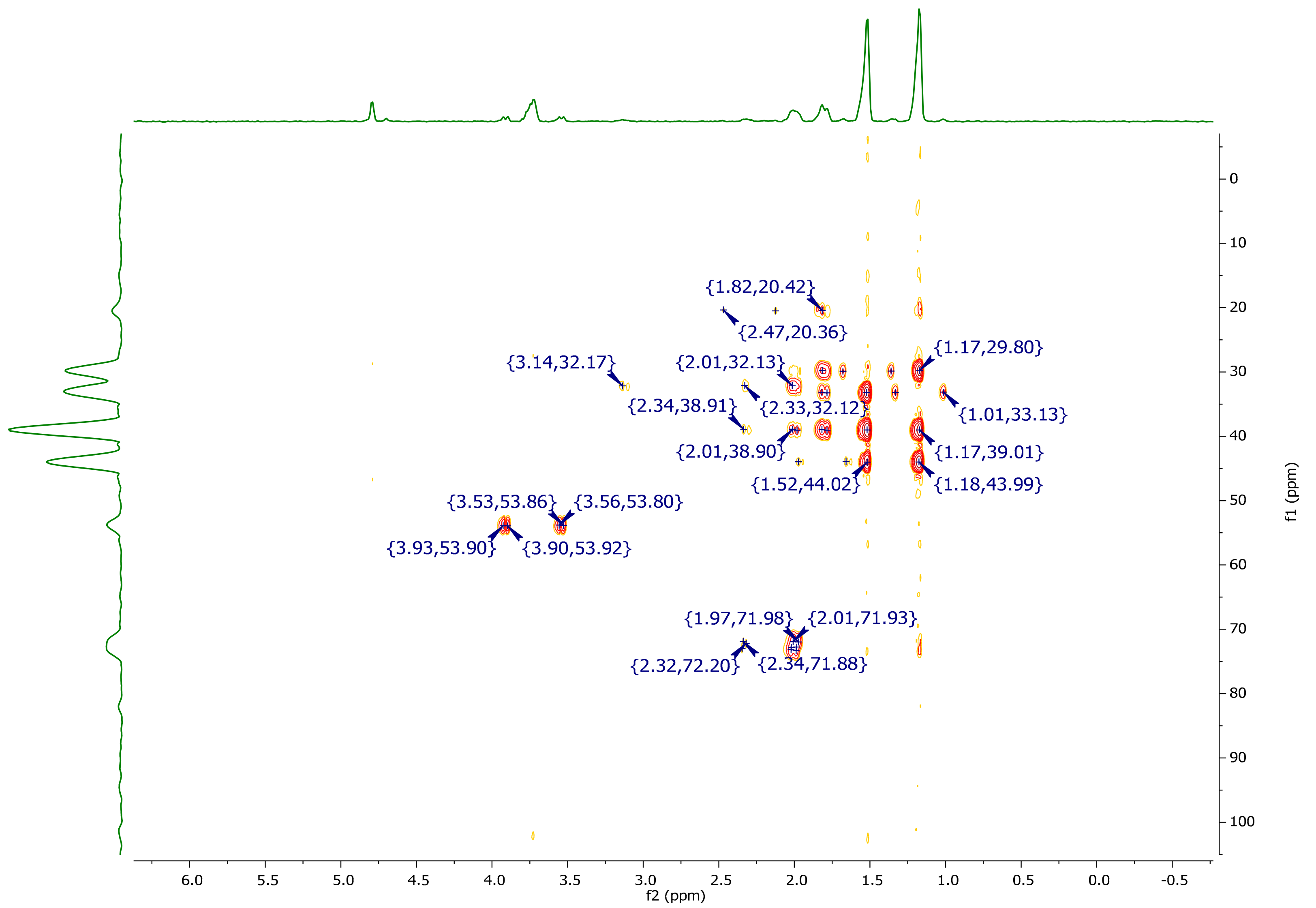

Figure S6. ^{1}H-^{13}C HMBC spectrum of dimethyl(4-hydroxy-2,2-dimethyltetrahydro-2H-thiopyran-4-yl)phosphonate.
